# Evaluation of the role of age in MMP 9 and MMP 2 levels in pulpal blood of teeth with symptomatic irreversible pulpitis

**DOI:** 10.1016/j.jobcr.2025.09.022

**Published:** 2025-09-27

**Authors:** Praneeth rednam, Sarath sarathy

**Affiliations:** a Department of Conservative Dentistry and Endodontics, Thai Moogambigai Dental College and Hospital, Chennai, India; b Department of Conservative Dentistry and Endodontics, Thai Moogambigai Dental College and Hospital, Chennai, India

## Abstract

**Aim:**

To assess MMP 2 and MMP 9 levels in pulpal blood of human molar teeth with symptomatic irreversible pulpitis in 2 different age groups.

**Materials and methods:**

Patients of two age groups young [18–40] and old [>60] with symptomatic irreversible pulpitis in molars were included in this study. Teeth were isolated using a rubber dam. The rubber dam, clamp, and tooth were decontaminated using a cotton swab drenched in 1 % NaOCl solution. Access cavities were then prepared using a diamond-coated bur in a contrangle handpiece under water-cooling. Subsequently, the caries was excavated using a sterile round bur. Pulpal blood was collected from the pulp chamber by placing the blunt end of a sterile inverted 60 size paper point for 1 min. The paper point was transferred into a sterile microcentrifugation tube and frozen at −20 °C immediately until further processing. The levels of MMP-9 and MMP-2 in these samples were measured using commercially available specific enzyme-linked immunosorbent assay (ELISA) kits. Data was analysed using an independent t-test.

**Results:**

The mean pulpal blood levels of MMP-9 in patients above 60 years (220 ng/ml) were significantly lower than those aged 18–40 years (468 ng/ml) ([p value < 0.05]

The mean pulpal blood levels of MMP-2 in patients above 60 years (134 ng/ml) were significantly lower than in those aged 18–40 years (153 ng/ml [p value < 0.05].

**Conclusion:**

MMP-9 and MMP-2 levels in pulpal blood of cariously exposed teeth with irreversible pulpitis can vary with age.

## 1 Introduction

The present understanding of pulp and the development of bioactive materials have led to a tremendous shift in the management of inflamed dental pulps.^1^ Pulpotomy can be better option for the management of teeth with partial irreversible pulpitis in contrast to root canal treatment.^3^

Pulpotomy is based on the concept that caries-exposed dental pulps react in a compartmentalised way to the invasion of bacteria and their by-products.^4^ The local inflammation and necrosis in pulp tissue progress slowly in an apical direction.^5^ After the amputation of infected coronal pulp, the residual healthy pulp tissue can heal if a viable environment is provided.^6^ The success rate of pulpotomy in mature permanent teeth with irreversible pulpitis ranges from 92 % to 100 %.^7^

Case selection criteria for pulpotomy are based on preoperative symptoms, clinical and radiographic signs, and intra-operative assessment of blood colour and volume and time needed to achieve haemostasis.^8^ However, these do not represent the biological status of the dental pulp. Histology is the gold standard for determining pulp inflammation status, but it can't be done when trying to maintain pulp vitality. Thus, the accurate diagnosis of pulp status remains difficult, and improper diagnosis often results in underestimating inflammation and finally lead to treatment failures.^1^^,^^10^

The progression of the process of inflammation from mild, moderate to severe inflammation is a result of the inflammatory mediator profile of dental pulp tissue. The molecular phase precedes the macroscopic and microscopic inflammatory changes.^1^ Hence, qualitative and quantitative assessment of these inflammatory mediators in pulpal blood can represent an objective method to know about the pulp status.

Matrix Metalloproteinases (MMPs) are zinc-dependent endopeptidases that cause degradation of extracellular matrix components, which occurs during normal and pathologic remodelling of tissues.^11^ Among the various MMPs^1^^,^^2^^,^^3^^,^^8^^,^^9^ studied in the dental pulp during inflammation, MMP-9 is considered very important in the breakdown of pulp tissue.^12^ The concentrations of MMP-9 are reported to be significantly different in pulp tissue during different stages of inflammation.^14^

A recent clinical trial highlighted the inflammatory predictive power of matrix metalloproteinase 9 (MMP-9; neutrophil gelatinase) levels in blood samples collected from the pulp on the treatment success of complete pulpotomy in permanent painful teeth.^15^ An important association was established between MMP-9 concentration and the outcome of pulpotomy procedures in mature permanent teeth with symptomatic partial irreversible pulpitis.^15^

MMP 2 [gelatinases or type IV collagenases] are also associated with neutrophil-related tissue breakdown in the pulp. MMP-2 expression in pulpal blood has been reported to show potential in the assessment of pulpal inflammation.^16^

However, there are no published studies evaluating the variations of MMP-9 and MMP-2 with age. Therefore, the present study aimed to investigate MMP-9 and MMP-2 levels in patients with symptomatic irreversible pulpitis (SIP) of two different age groups. The null hypothesis evaluated was that there would be no difference between MMP9 and the MMP-2 levels of inflamed pulpal blood in young and older patients.

## 2 Materials and methods

The study was reviewed by the Institutional Review Board of DR. MGR Educational and Research Institute [Deemed to be a university], Maduravoyal, Chennai. [NO.221/2024/IEC/TMDCH] and --- The study was registered in the Clinical Trials Registry of India [CTRI/2024/10/076026]. Patients were recruited from those referred to the Department of Conservative and Endodontic for pain management. Patients with a diagnosis of symptomatic irreversible pulpitis were identified. Next, informed patient consent was obtained, and then patients were included in the study based on below inclusion and exclusion criteria.

## 3 Inclusion criteria

Patients of age [20–40yrs], young and old [above 60yrs] with symptomatic irreversible pulpitis in molars were included. Characteristics included sharp pain upon thermal stimulus, lingering pain (30 s or longer after stimulus removal), spontaneity (unprovoked pain), and referred pain-AAE. Patients with both class one and class two cavities were included.

## 4 Exclusion criteria

Patients with known systemic diseases as diabetes and hypertension, patients who have taken NSAIDS, pregnant and lactating women, patients with a diagnosis of reversible pulpitis, and patients with cracked teeth and non-vital teeth were excluded from this study.

### 4.1 Sample size calculation

The sample size was calculated by determining the effect based on the data from a pilot study comprising 5 patients, using G Power v3.1 software [University of Dusseldorf, Germany]. The required sample size to obtain significant results in the MMP9 group is 8, and MMP2 is 4.

### 4.2 Clinical procedure

Local anesthesia (2 % lidocaine hydrochloride with epinephrine, 1:80,000; was given, and teeth were isolated with a rubber dam (Coltène/Whaledent Inc). Rubber dam wiped with 3 % hypochlorite for disinfection.^17^ Cavities were prepared with a round bur BR-49 with diameter 0.5 mm (MANI inc, Japan) in a contra-angle handpiece under constant water cooling. The caries was completely (nonselectively) removed from all of the cavities, and excavation continued until the dentin was hard.^17^

Bleeding from the exposed pulp was controlled by pressing a cotton pellet soaked in sterile physiologic saline against the wound for 1 min. Subsequently, pulpal blood was collected with an inverted size 60 sterile paper point for 1 min (Diadent, USA) for MMP2 and 9 analysis.^18^

## 5 MMP 2 and MMP9 level calculation

Paper points (1 per case) were collected in individual sterile microcentrifugation tubes and stored at −20 °C until analysis.^18^ On the day of analysis, the samples were eluted in 2 ml of sterile phosphate-buffered saline (pH 7.2) by centrifuging at 2000×*g* for 30 min at 4 °C. The supernatant was collected and used for analysis. The levels of MMP-9 and MMP-2 were measured with a commercially available specific enzyme-linked immunosorbent assay kit (Quantikine ELISA; R&D Systems). The detection range of the assay is 31 to 2000 ng/ml.

### 5.1 Statistics methods

MMP2 and MMP9 values were compared between young and old patients and also within each group [old or young] using an independent sample *t*-test. A p-value less than 0.05 was considered statistically significant.

### 5.2 Results

20 patients met the inclusion criteria and were categorized into two groups: old [n = 10] and young [n = 10]. Mean values of MMP9 and MMP2 were calculated for each group and were compared (Table 1). MMP9 and MMP2 levels were significantly higher in young individuals with irreversible pulpitis than in the older age group (P = 0.001, P = 0.001).

**Table 1:** Comparison of MMP2 and MMP9 between young and old patients using an independent sample *t*-test.

	Age	N	Mean	Std. Deviation	Test statistic	P value
MMP2	Young	10	157.48	3.04	t = 14.527	0.001∗
	Old	10	134.82	2.79		
MMP9	Young	10	469.27	74.82	t = 5.587	0.001∗
	Old	10	223.21	89.32		

p ≤ 0.05 is considered statistically significant.

In both younger and older individuals, MMP-9 values were significantly higher than MMP-2 [Table 2, Table 3]. [P = 0.001, P = 0.023.]

**Figure:**
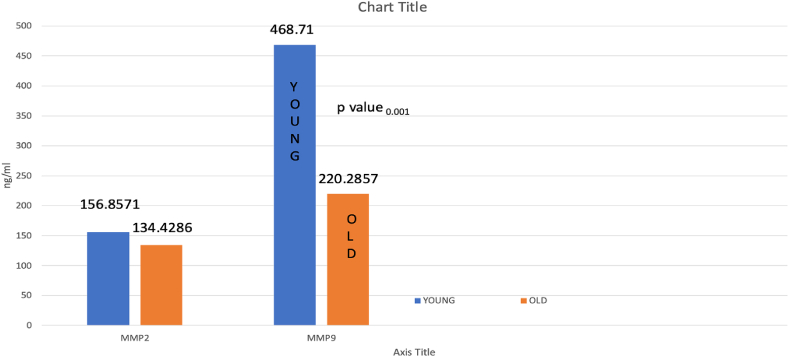

**Table 2:** Comparison of MMP 2 and MMP 9 in young patients using an independent sample t-test.

Protein	N	Mean	Std. Deviation	Test statistic	P value
MMP 2	10	157.48	3.04	t = −11.016	0.001∗
MMP 9	10	469.27	74.82		

p ≤ 0.05 considered statistically significant.

**Table 3:** Comparison of MMP 2 and MMP 9 in old patients using independent sample *t*-test.

Protein	N	Mean	Std. Deviation	Test statistic	P value
MMP 2	10	134.82	2.79	t = −2.617	0.023∗
MMP 9	10	223.21	89.32		

p ≤ 0.05 considered statistically significant.

## 6 Discussion

The status of pulpal inflammation has been divided into reversible and irreversible based on pain history and symptoms (AAE). Irreversible pulpitis " itself implies that the pulp will not heal.^2^ So, the use of the term “irreversible” is challenged as it underplays pulp's healing potential and may lead to pulpectomy or tooth extraction.^19^ Recent literature has reported that pulpotomy in teeth with partial irreversible pulpitis has a high success rate, and that the outcome was comparable to root canal treatment.^7^

Objective identification of the extent and severity of pulpal inflammation can increase the success rate of pulpotomy.^7^^,^^19^ An objective molecular diagnostic method is necessary to address the shortcomings in the present pulpal diagnosis. Various inflammatory mediators (immunoglobulins, cytokines, prostaglandins, proteases, etc.) are studied to differentiate between inflammatory and non-inflammatory conditions of the dental pulp.^1^ Increased expression of substance P, neurokinin A, and MMP-9 is seen in the gingival crevicular fluid (GCF) of teeth with irreversible pulpitis.^8^ The levels of FGF acid, IL-6, IL-1a, TIMP-1, and MMP-9 in dentinal fluid (DF) can help differentiate healthy and inflamed pulp.^20^

The chemical analysis of GCF or DF could be a useful non-invasive diagnostic tool to make key decisions for the management of cases of deep carious lesions. However, in teeth with carious exposure, the pulpal blood sample gives the site-specific pathophysiologic condition of the dental pulp.^20^ A significantly higher level of IgG, IgA, IgM, elastase, prostaglandin E2, Interleukin 6,8,10, TNF-α, IFN-γ, and MMP-9 were detected in caries-exposed pulps and irreversible pulpitis [IRP] as compared to pulps of normal teeth.^13^^,^^21^ So, pulpal blood was chosen for investigation in the present study.

MMP-9 and MMP-2 [gelatinases or type IV collagenases] have been associated with neutrophil-related tissue breakdown in the pulp. MMP-9 was more extensively studied, identifying it as an important gene for pulpal inflammatory status.^17^^,^^18^ MMP-9 was higher in teeth with IRP as compared to healthy controls.^14^ On the other hand, MMP-2 also increases with inflammation.^17^^,^^22^ However, the effect of age on MMP-9 and 2 in pulpal blood has not been extensively studied.

Therefore, our study aims to evaluate MMP-9 and MMP-2 in pulpal blood of young and old patients with SIP. Zymography, calorimetric ELISA, and fluorometric assay were the commonly used techniques in clinical research for the determination of MMP-9 and its activity.^23^ Zymography is a highly sensitive method to detect the activity of MMP-9, but it does not quantify MMP-9.^24^ The majority of MMP-9 is in a latent form, but it is the active form that causes biological activity. Conventional ELISA and fluorometric assay can identify both active and pro-form of MMP-9. So, conventional ELISA was used in the present study to quantify MMP levels.

The volume of the collected sample must be adequate for repeated and accurate measurement of the concentration of biomolecules in an assay. In previous studies, the pulpal blood samples were collected using cotton pellets, capillary tubes, and perio paper strips, resulting in a small volume collection^18^ In the present study, pulpal blood was collected using a paper point, because it has the advantages of precise localization, sterility & reduced contamination, controlled volume, efficient handling & transport, and cost-effectiveness^17^

The results of this study showed higher mean MMP-9 values in pulpal blood of young individuals than older individuals due to an active inflammatory pathway [Young-469 ng/ml vs old-223 ng/ml]. Hence, our null hypothesis was rejected. In irreversible pulpitis, MMP-9 facilitates the breakdown of the basement membrane and ECM, allowing immune cells to infiltrate the inflamed pulp tissue.^25^

Young individuals exhibit higher MMP-2 levels due to their greater regenerative potential, which is seen in our findings. [Young-157 ng/ml vs Old 134 ng/ml]. MMP-2 is involved in tissue remodelling and repair. It is normally expressed in healthy pulp, but it is increased during inflammation.^10^ In irreversible pulpitis, MMP-2 contributes to the degradation of collagen and other ECM components, helping tissue reformation. Aggressive inflammatory response produces more MMP9 than MMP 2, which explains more MMP-9 levels [469 ng/ml] than MMP-2 [157 ng/ml] in our findings in young pulp, and the same applies to older pulp.^26^

High MMP-9 and 2 levels in younger people can be explained by that young pulp is highly vascularized, cellular, and metabolically active. This results in a more robust inflammatory response to injury or infection, such as in irreversible pulpitis.^13^ MMP-9 (gelatinase B) and MMP-2 (gelatinase A) are key enzymes involved in degrading extracellular matrix (ECM) components, facilitating immune cell migration, and mediating tissue remodelling. Also, their increase in young pulp reflects the tissue's attempt to repair and respond to inflammation.^27^ The increased inflammatory response in young pulp leads to increased production of pro-inflammatory cytokines (e.g., IL-1β, TNF-α), which in turn stimulate MMP-9 and MMP-2 expression.^28^] The presence of more stem cells and progenitor cells in young pulp also leads to higher MMP activity, as these cells actively participate in tissue repair and regeneration^29^

The low MMP 9 and 2 values in the old cohort are explained as follows: with age, the pulp undergoes degenerative changes, including reduced cellularity, decreased vascularity, and increased fibrosis. These changes lead to a very minimal inflammatory response and lower metabolic activity.^29^ As a result, MMP-9 and MMP-2 levels can be lower in older individuals, even in cases of irreversible pulpitis, signifying reduced tissue reaction and regeneration potential.

Also, age-related changes, such as reduced cellularity and vascularity, result in a reduced inflammatory response and lower cytokine production, leading to decreased MMP-9 and MMP-2 levels. The accumulation of cross-linked collagen and other ECM components in older pulp further reduces the activity of MMPs, interfering with tissue remodelling^25^ Older patients (over 60 years old) have a delayed pulp response and reduced pain intensity compared with younger patients due to calcifications Pulp space diminishes through life by the deposition of secondary dentin During the aging process, there was not only a decrease in the size of the pulpal chamber but also a loss of blood vessels and nerves. Also, the inflammatory mediator pathway of MMP can be disturbed in older age populations^28^

**Clinical Implications**: Higher MMP-9 and MMP-2 in young individuals with irreversible pulpitis may serve as biomarkers for more aggressive disease progression and a greater need for timely intervention.^29^ In older individuals, lower MMP9 and 2 levels may indicate a similar disease but also a reduced capacity for healing, necessitating different treatment strategies.^30^**.**

Further research is needed to elucidate the precise mechanisms underlying age-related differences in MMP-9 and MMP-2 expression in irreversible pulpitis. Longitudinal studies could explore how MMP levels change over time and their correlation with treatment outcomes in different age groups. MMP levels may vary not only with age but also by gender and systemic diseases in inflamed pulp, which need further long-term research to be identified as a viable biomarker. Also, not only MMPs, other potential biomarker molecules such as IL-8, IL-6, TNFα, IL1β, FGF, and VEGF-α, should be looked at in the pulpal blood of young and old patients. Furthermore, studies are needed to investigate MMP9 and MMP-2 in the pulpal blood of young and old patients to verify the results of the study.

## 7 Conclusion

Within the limitations of this study, the mean MMP-9 and MMP-2 levels in pulpal blood of patients above 60 years were significantly lower than in those aged 18–40 years. The MMP9/2 ratio in patients aged 60 years was 1.75, while it was significantly higher in patients aged 18–40 years, which was 3.02.

## Funding

This research did not receive any specific grant from funding agencies in the public, commercial, or not-for-profit sectors.

## Declaration of competing interest

The authors declare that they have no known competing financial interests or personal relationships that could have appeared to influence the work reported in this paper.
